# Financial Incentives to Stimulate Integration of Care

**DOI:** 10.5334/ijic.2532

**Published:** 2016-10-28

**Authors:** Apostolos Tsiachristas

**Affiliations:** Health Economics Research Centre, Nuffield Department of Population Health, University of Oxford, GB

## Introduction

Chronic conditions are related to 60% of disability adjusted life years and account for 75% of total health care expenditure worldwide. The economic burden of chronic conditions is much larger when considering the costs of productivity loss and informal care. The threat that chronic conditions pose to population health and economies increase because of increasing prevalence and multi-morbidity, which multiplies the burden of chronic conditions. Integrated care is seen as the means to tackle this threat by improving population health and patient experience with care, and reducing costs. It includes initiatives that seek to improve outcomes for those with (complex) chronic health problems and needs by overcoming issues of fragmentation through linkage or coordination of services of different providers along the continuum of care. In the era of personalized medicine and genomics, integrated care may be characterized by the recently suggested term ‘humanomics’ because it incorporates treatment based on personal need, preferences and capacity, it interacts with the context in which it is implemented, and its success depends highly on human behaviour [1]. Financial incentives are one of the main prerequisites for integrating care [2].

## Financial incentives

There are different types of incentives at different levels of the healthcare system targeting different stakeholders. The four main categories of incentives targeting healthcare professionals include financial incentives, professional ethics including intrinsic motivations, organizational cultures for example informal behavioural codes, and policies and governance. Financial incentives may take the form of rewards or penalizations to inspire and motivate individuals and organizations to work towards defined objectives – usually in a contractual relationship. However the goal of financial incentives is more than just rewarding good performance and punishing bad performance. Financial incentives can support the change of current health and social care delivery by stimulating both immediate and long-term improvements in performance, aligning expectations and rewards, and removing financial barriers that perversely effect desired performance. However, financial incentives should be used with cautiousness because they may undermine intrinsic motivations. Especially in health and social care, where intrinsic motivations play an important role at individual and organizational level, financial incentives may even crowd-out intrinsic motivators such as purpose and altruism. This debate is reflected in the attempt of Glasziou and colleagues to develop a checklist about whether financial incentives via bonuses would be beneficial rather than harmful [3].

But is financial self-interest wrong by nature and contradicts with intrinsic motivation? Thousands of years ago, the first medical physicians in ancient Greece had interest in money to make a living and they were publicly admitting it. The medical ethics in that time, ordained that physicians should not be preoccupied by money, not that physicians should be indifferent to money. So financial incentives are compatible with medical codes. Another questions is, is financial self-interest wrong when combined with competition? Classical economist and philosopher Adam Smith argued in the 18th century that if the self-interest of suppliers was matched by the self-interest of consumers that would lead to an optimal outcome for society, as if an invisible hand leads people to act in ways which were not part of their original intention. In the health and social care sector, which is characterized by several market failures, governments (mainly in Europe) took-up the role of matching self-interests and became the visible hand leading individuals and organizations to achieve optimal outcome for the society. So financial incentives can be combined with elements of competition and governmental regulation to maximize welfare.

## Financial incentives to stimulate integration

The problem with traditional payment schemes is that they do not provide adequate financial incentives to integrate care. Salary fails to stimulate integration of care because there are potential incentives to accept only healthy patients (cream skimming) and to refer complex cases to more costly secondary services (dumping). Capitation provides caregivers with an incentive to spend a little amount of time on each patient such that more patients can be enrolled that generate compensation. As such, chronically ill are financially unattractive as they require more time and services to treat, at the expense of the physician, who would otherwise receive the same remuneration for treating a healthier patient who merely requires an occasional simple, quick treatment. Fee-for-service (FFS), on the other hand, generates an incentive to provide as many refundable services as possible. While FFS reduces the incentive to avoid the chronically ill, there is little incentive for caregivers to provide high quality of care and adequately address the needs of patients with chronic diseases.

For this reason, alternative financial arrangements have been developed to incentivize integration because evidently, integrated care requires integrated payment.

**Figure 1:**
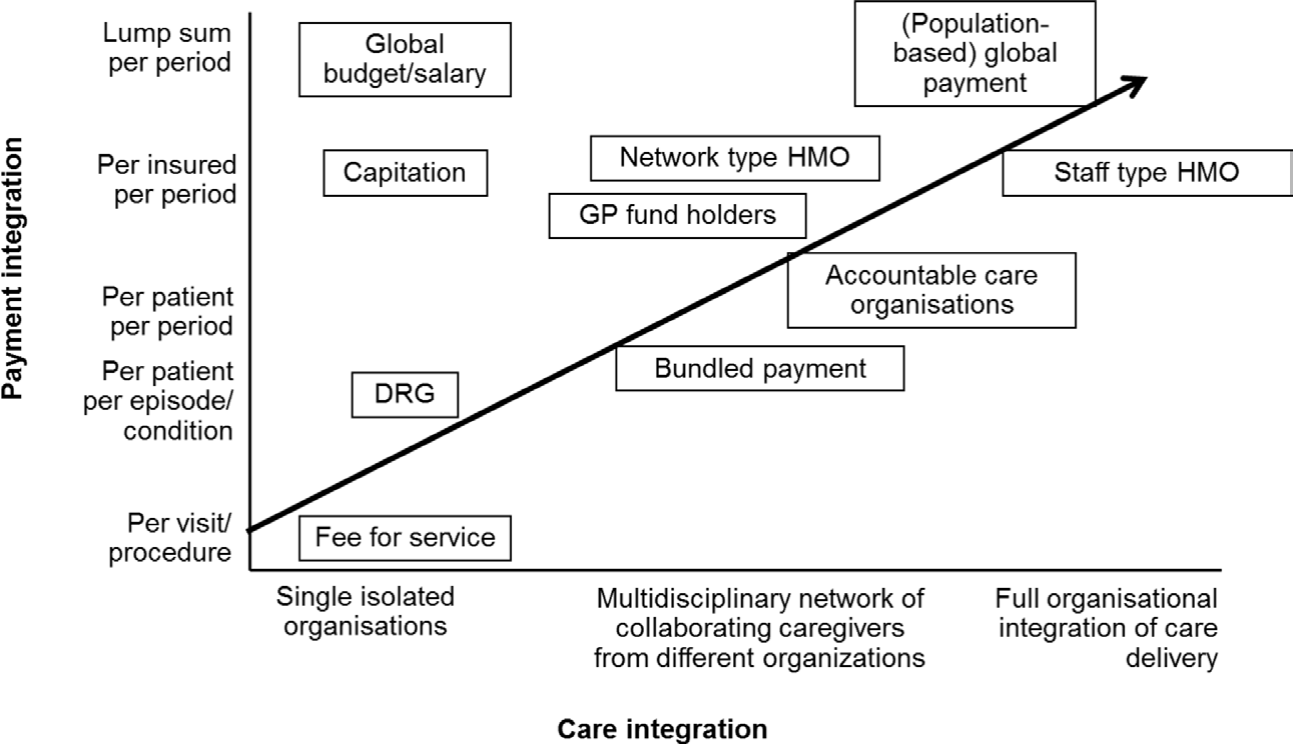
Payments alongside integration of care. (the figure is based on Shih al. The Commonwealth Fund 2008 & Eijkenaar et al Eur J Health Econ 2013; 14: 117–31 and adjusted by Maureen Rutten-van Mölken and the author).

In Figure 1, different payment methods are plotted according to their level comprehensiveness and scope to integrate care. For example, the traditional FFS, which is used to reimburse single isolated organizations per provided service, is located on the lower left corner of the figure, while a population based global payment, which reimburses all care needed from a specific population for a time period, is located on the upper right corner. However, ‘comprehensive’ or ‘integrated’ payments are associated with higher financial risk, which in turn could be a disincentive to move towards such payments. This disincentive maybe a large barrier to design adequate payments for integrated care as the financial risk will be distributed differently between payers and providers and can cause therefore, major opposition. For this reason, we need to incentivize the major stakeholders involved in the integration of care. Incentives for purchasers/payers, which apply in particular in the context of health systems with a clear purchaser-provider split and/or the presence of various purchasers, have been designed to steer the allocation of resources towards coordination and more integrated care delivery. An example of such incentives is the ‘Accountable Care Organizations’ with population based payment or earmarked payments for participating in specific disease management programs. The bulk of incentives for integrated care is linked to paying providers and reaches from ‘aligned budgets’, ‘pooled funds’ or ‘bundled payments’ for care groups in the context of disease management programs to ‘pay-for-coordination’ and various other mechanisms along the ‘value-based’ payment continuum [4]. Patients, as co-producers of outcomes, may be incentivized in particular with respect to patient compliance concerning treatment plans and medication, e.g. by means of personal health budgets or waivers/reductions of out-of-pocket contributions. Such incentives usually are supported by non-financial incentives concerning preventive and health promoting measures such as discount for gym membership, privileged access to physicians outside normal hours or general measures to improve health literacy. The most important aspect in incentivizing different stakeholders is to align the incentives across them!

Most of the alternative financial agreements presented in Figure 1 were designed and implemented by purchasers of health care in the U.S. to contain costs and improve quality of care. The most well-known value-based financial agreements implemented in the U.S. are global payment, accountable care organizations with shared savings program, pay-for-performance (PFP), bundled payment, and pay-for-coordination (PFC). These schemes are not just an alternative method for paying health care providers. They introduced financial risk to providers, explicit measures of quality improvement driven by financial incentives to providers, efforts towards patient-centred care through integration and coordination of care, and financial incentives for patient safety.

These alternative payment schemes were transferred to Europe after being adapted to the European context and social values and were accompanied by broader reforms in health and social care systems. The adoption and adaptation of alternative financial agreements was based on the main features of a health care system to save time and effort as well as to successfully implement them. In many European countries alternative payments were combined (for example PFC and global payment in Germany) or provided on top of traditional payments (e.g. PFP on top of capitation and FFS in England), targeted key-stakeholders that were expected to respond to the financial incentives, had different form and size, incentivized the integration of different services, and their uptake varied across countries [5,6]. Some countries, such Austria, Denmark, France, and Germany, introduced pay-for-coordination to reward the coordination of multidisciplinary care teams. Other countries, including England and France, introduced PFP to reward improvements in process and outcomes of care. Bundled payment for a group of services for a specific disease involving multiple providers for a fixed time period was introduced in The Netherlands. Global payment in a form of a retrospective risk-adjusted payment for a full range of services related to a specified group of people was introduced in Germany. These financial agreements were chosen by policy-makers to be implemented because positive evidence from the implementation of these financial agreements in the US was reported in the literature [7,8,9,10,11,12].

## Facilitators, barriers and impact of financial incentives

A recent study found that the most frequent facilitators for the successful implementation of these financial agreements were a strong cooperation between stakeholders and the adequacy of the provided financial incentives to key-stakeholders. On the other hand, it was found that gaming of the payment mechanism and misaligned incentives between stakeholders were the most frequent barriers [5]. Another study investigated the impact of the financial agreements introduced in Europe on health care expenditure and found that PFC, bundled payment and global payment reduced the growth of outpatient expenditure at the year of implementation while PFP reduced the growth of hospital and administrative expenditure at the same year [6]. However, the impact on health care expenditure was volatile in the 4-years after implementation and only PFP, bundled payment and global payment had a sustainable negative effect on health care expenditure growth. The concerns raised by this study were that PFC is suitable merely as start-up payment for integration, PFP may jeopardize the quality of non-rewarded services, and bundled payment and global payments may cause supply induced demand. Based on these findings, we favoured a blended payment scheme with a yearly risk-adjusted population-based global payment as basis, supplemented by pay-for-coordination and pay-for-performance. In this blended payment, shared savings to avoid “gaming”, align incentives, support prevention, and reward patients may be also used. Irrespective of how a health care system is funded, policy makers are still trying to find appropriate models of providing financial incentives to integrate care. A substantial start-up funding to integrate care across sectors was recently introduced via the Health care Strengthening Act in Germany, a country with a social insurance based healthcare system. Similar, a pooled budget to integrate health and social care, namely the Beter Care Fund, was introduced in England, a country with a tax-based financed system. In addition, the year of care, a capitated payment for a broad range of services for a defined time period was also introduced in England. The Netherlands, with a mix of tax and private insurance based system, experiments with population-based global payments including pay-for-performance and shared savings while as of last year, primary care is reimbursed via a 3-tier system that combines traditional payment (i.e. capitation and FFS) with PFC and PFP.

## Designing and implementing financial incentives

Irrespective of the chosen financial agreements, designers of financial incentives to stimulate integration of care should: 1) come-up with comprehensive, evidence-based incentives aligned with intrinsic motivations, 2) reward risk with premium, 3) find a balance of rewards and penalties depending on the context, 4) offer stakeholders with a choice of financial incentives as different individuals respond differently to incentives, 5) find an optimal blend of group and individual level incentives, 6) combine absolute with relative targets differentiated across groups, 7) define the right incentive size and make it known in advance, 8) minimize the elapsed-time between provision of integrated care and reward, and 9) find incentive with sustainable effect in time. Furthermore, the successful implementation of financial incentives for integrated care requires: 1) a clearly defined population, 2) sufficient and relevant data to compensate for high risk patients, 3) unambiguous and measurable goals to determine success, 4) broadly accepted, sensitive, and clinically relevant indicators, 5) transparency and willingness to record results, 6) involved parties share commitment and goals, 7) insight into costs of the population, 8) high degree of organization in primary care, 9) an integrated ICT system, and 10) long-term scope with sufficient time allowed to wait for the first concrete evidence.

Related to the last point, decision-makers need thorough and periodic evaluations of financial incentives. For this reason, more rigorous study designs are needed to account for the selection of physicians into incentive schemes, disentangle the effect of simultaneously implemented changes in health care and infer causality, and examine the potential unintended consequences of financial incentives. In addition, studies should describe more consistently the type of financial agreement at baseline or in the control group as well as the size of the financial incentives and how they were used and distributed. Further research should focus on finding optimal mixture (type and size) of financial incentives to stimulate integration of care and compare financial incentives with other behaviour change interventions.

## Conclusions

Financial incentives are potentially powerful tools to stimulate integration of care. Policy-makers should use them as means to extend the cost-effective potential of integrated care rather than as cost-containment policies. They should also use experiences from other countries with comparable healthcare systems and context. Finally, they should have strong willingness and commitment because repositioning financial incentives and changing behaviour in the healthcare sector is not an easy task.

## Author Information

Apostolos is a senior researcher at the Health Economics Research Centre, University of Oxford. His main research interests are related to the financing and economic evaluation of complex interventions, with a particular focus on integrated care. Apostolos’ work has been published in scientific journals and presented in international conferences. He is also associate editor in international peer-reviewed journals and leads the Health Economics in Integrated Care Special Interest Group (HEIC-SIG) of the International Foundation for Integrated Care (IFIC).
